# Single-cell RNA sequencing reveals microglial proliferative bias and neuroinflammatory communication reprogramming following traumatic brain injury

**DOI:** 10.3389/fneur.2026.1789863

**Published:** 2026-07-10

**Authors:** Xue Zhang, Na Sun, Manman Zhu, Yan Huang

**Affiliations:** The First Affiliated Hospital of Anhui University of Science and Technology, Anhui, China

**Keywords:** cell communication, cortical impact, M1 phenotype, microglia, neuroinflammation, proliferation, single-cell RNA sequencing, traumatic brain injury

## Abstract

Traumatic brain injury (TBI) remains a major global health challenge, with complex and incompletely understood pathophysiological mechanisms. In this study, single-cell RNA sequencing was employed to systematically characterize the transcriptional landscape of cortical cells in the mice model of moderate controlled cortical impact. Analysis of three TBI samples and three sham samples identified 14 different cell groups. In particular, seven microglial subclusters were identified, revealing significant phenotype remodeling of microglial. After TBI, the homeostatic microglial subclusters (C0 and C2) were reduced, whereas disease-association subclusters (C1, C4, and C5) and a proliferation subgroup (MC Cycle) were markedly increased. Pseudotime trajectory analysis further confirmed the transition of microglial subclusters from a homeostatic state to a pro-inflammatory state, characterized by the downregulation of Tmem119 and the upregulation of Tnf, Spp1, Il1a, Il1b, and Cxcl2. Moreover, proliferating microglial in the TBI group predominantly exhibited an M1-like phenotype. Intercellular communication analysis revealed a substantial reconstruction of a cellular interaction network. Notably, microglia enhanced signal transmission through specific pathways, thereby promoting M1 polarization, contributing to barrier dysfunction and mediating neuroinflammation responses. Overall, our findings provide a high-resolution map of cellular dynamics of TBI posterior cortical cells, highlighting the preferential shift of the regeneration of microglial toward a pro-inflammatory phenotype, and revealing a complex multicellular communication network centered on M1-like microglia.

## Introduction

Traumatic brain injury (TBI) represents a major global health challenge and contributes substantially to mortality and long-term disability across different populations (1, 2). Epidemiological studies indicate that TBI affects approximately 50–74 million individuals worldwide every year (3, 4). It is also recognized as one of the leading causes of death among the younger individuals, especially those under 45 years of age (5, 6). In addition to its clinical impact, TBI imposes a considerable economic burden with annual global costs estimated of hundreds of billions of dollars, accounting for nearly 0.5% of the world's gross domestic product (5). There is a heterogeneous spectrum of TBI in clinical manifestations, ranging from mild concussion to severe traumas (2). Although prolonged therapeutic interventions have been shown to improve long-term neurological outcomes, effective treatment for TBI as a chronic health condition remains limited. Current clinical management mainly focuses on acute surgical and supportive therapy, including intracranial pressure control and tubular edema. However no pharmacological therapies for moderate or severe TBI have been successful translated into clinical trials (7–9). This therapeutic limitation reflect the complex pathophysiology of TBI and highlight the urgent need for a deeper mechanistic understanding to identify novel therapeutic targets. The cerebral cortex is particularly vulnerable to traumatic injury, in both focal and diffuse forms of TBI. Primary injury commonly involves direct mechanical damage, including contusions, hematomas, and immediate disruption of neural tissues and vessels (2, 6, 10). However, the progression of TBI is mostly driven by secondary injury processes that may develop over minutes to months after the initial injury. These secondary pathological events including excitotoxicity, oxidative stress, metabolic disturbances, and neuroinflammation are prominent within the cortex and contribute to delayed cellular damage and tissue degeneration (11–14). Structural imaging and histopathological studies in both human beings and animal models have demonstrated cortical thinning, gray matter loss and traumatic axonal injury following TBI (15, 16). Such changes do not occur in a vacuum longitudinal changes demonstrate continuing cortical atrophy, in terms of cortical thickness and area, and may extend across years, and be associated with sustained cognitive and functional losses. This progressive cortical degeneration emphasize that TBI should be regarded as a chronic disease process and underscores the important of investigating the cellular and molecular mechanisms underlying these structural changes.

The central component of secondary injury is the robust neuroinflammatory response, which is primarily regulated by resident glial cells; particularly microglia and astrocytes (12, 17, 18). Microglia the innate immune sentinels of the central nervous system, are among the earliest cells to respond to brain injury. During the acute phase, they undergo rapid morphological changes, proliferation, and migration toward the site of injury. This activation is facilitated by multiple signaling pathway, including purinergic receptors and damage-associated molecular patterns (19–21). Microglial responses exhibit a dual nature; on one hand, they may represent pro-inflammatory (M1-like) responses releasing cytokines into the tissue, such as TNF-a, IL-1b together with reactive oxygen species, on the other hand, microglia can assume an anti-inflammatory (M2-like) phenotypes, which rehabilitate the tissue by repair and resolution effectors (22, 23). In TBI, however, this balance is often disrupted, resulting in prolonged inflammation activity. Chronically activated or primed microglia become high responsive to subsequent immune challenge, which can further increase neurodegeneration (24). These persistent, long-term microglia responses have been observed in both animal models and human patients with TBI and are associated with continued tissue damage and functional impairment a process that may ultimately contribute to progressive neurological disability (23). Astrocytes, the most abundant glial cell type in the central nervous system, also play important roles in pathogenesis TBI. Astrocytes closely interact with microglia and vascular cells, and are involved in maintaining blood-brain barrier integrity, synaptic function and inflammatory signaling (25–27). Dysfunctional astrocytic responses may impair glutamate clearance, enhance excitotoxicity and disrupt neuronal networks, ultimately contributing to neuronal damage and disease progression (28).

In recent years, single-cell RNA sequencing (scRNA-seq) has emerged as a powerful approach for investigating the cellular complexity and heterogeneity of cortical responses in TBI. By enabling high resolution gene expression at individual cell level, scRNA-seq has allowed researchers to characterize distinct cell populations, characterize novel cellular subtypes, and detect cell-specific transcriptional alterations that cannot be resolved through conventional bulk tissue analyses. In the context of TBI, scRNA-seq studies have revealed cell type's specific responses involving in microglia and astrocyte subpopulations, endothelial cells, neurons and other glial cell types (29–31). However, the existing TBI datasets have not been fully explored with respect to the detailed- organization of microglial sub-lineages and their complex intercellular communication networks.

In summary, this work provides a refined reanalysis of the mammalian cerebral cortex atlas following moderate controlled cortical impact (CCI) model. Through the integration of advanced trajectory analysis and cell-to-cell communication modeling, we identified previously unrecognized proliferative dynamics of microglial as well as novel signaling pathways—including the OSM-LIFR and CD80–CD274 pathways—which were not reported in the primary analysis of this dataset. These findings provide additional mechanistic insights into the transition from acute injury to chronic neuroinflammation and identify potential molecular targets for future immunomodulatory therapeutic strategies.

## Methods

### Single-cell RNA sequencing data acquisition and preprocessing

Publicly available single-cell RNA sequencing (scRNA-seq) data derived from the cerebral cortex of a traumatic brain injury (TBI) mouse model were obtained from the Gene Expression Omnibus (GEO) database under accession number GSE290150. Briefly, the study utilized male C57BL/6 mice aged 2 to 3-month. To maintain analytical consistency and focus, only the normothermia (37 C) sham and TBI groups were included in the present analysis, whereas hypothermia-treated samples were excluded. Following anesthesia (Ketamine 100 mg/kg; Xylazine 10 mg/kg), a 4 mm diameter craniotomy was performed 0.5 mm to the midline between lambda and bregma. Moderate traumatic brain injury (TBI) was induced using a Controlled Cortical Contusion (CCI) device (Custom Design and Fabrication, Richmond VA) at a velocity of 4 m/s, with an impact depth of 0.8 mm for a 150 ms duration. Post-operatively analgesia was administered using buprenorphine (0.1 mg/kg). Sham-operated animals underwent identical surgical preparations and pharmacological treatment procedures but did not receive craniotomy or cortical impact injury. All CCI and Sham animals were sacrificed 24 h after injury for downstream analysis. Brain tissues samples used for single-cell RNA sequencing were carefully collected from the cortical injury site and the surrounding penumbra area.

Raw gene expression count matrices were processed and analyzed using the Seurat package (v4.0) in R. Rigorous quality control (QC) procedures was applied to filter out low-quality cells and genes. Cells expressing fewer than 200 genes, exhibiting hemoglobin gene content exceeding 1%, or showing mitochondrial gene content exceeding 20% were excluded to reduce the impact of technical artifacts and apoptotic cells. Potential doublets were identified and removed using DoubletFinder (v2.0.3) with default parameters. Following quality control, only high quality cells were retained for subsequent analyses.

### Single-cell RNA sequencing data integration

To integrate scRNA-seq data across multiple samples and batches, Harmony (v0.1.0) was applied using the RunHarmony function with over 10 iterations with default theta and lambda parameters. Additional technical variations, including differences in sequencing depth, were regressed out using the ScaleData function before downstream analysis.

### Dimensionality reduction, clustering, and visualization

Principal component analysis (PCA) was conducted on scaled data using highly variable genes (HVGs). Based on an elbow plot and cumulative explained variance, the top 30 principal components (PCs) were selected for downstream analyses. Cell clustering was performed using the FindNeighbors function based on the top 30 PCs, followed by clustering with the FindClusters function. To determine the optimal clustering resolution, multiple values ranging from 0.1 to 1.0 were evaluated. A resolution of 0.3 was selected as it provided biologically meaningful clustering with distinct transcriptomic signatures without over-fragmentation. For visualization, Uniform Manifold Approximation and Projection (UMAP) was performed using the same 30 PCs to generate two-dimensional embeddings. Cell clusters were annotated according to the expression patterns of well-established marker genes reported in previous studies.

### Differential gene expression and functional enrichment analysis

Differentially expressed genes (DEGs) between clusters were identified using the FindAllMarkers function in Seurat. Genes were considered significant if they met the following criteria: absolute log-fold change (|logFC|) ≥ 0.25, minimum fraction of cells expressing the gene (min.pct) = 0.25, and an adjusted *p*-value < 0.05 (Benjamini–Hochberg correction). Gene Ontology (GO) enrichment analysis of DEGs was performed using the clusterProfiler package (v4.2.2), with terms considered significantly enriched at *p* < 0.05.

### Microglia subpopulation analysis

Microglia were extracted from the integrated dataset based on canonical marker expression. The microglial subset was processed independently following the same analytical workflow, including normalization, HVG selection, PCA, and clustering. Cell–cell neighborhood relationships and cluster assignments were recalculated using the FindNeighbors and FindClusters functions with a resolution of 0.3 to define distinct microglial subpopulations.

### Pseudotemporal trajectory analysis

To investigate the developmental and activation trajectories of microglia subpopulations, pseudotemporal ordering was conducted using Monocle2 (v2.22.0). The microglial subset was first processed to identify ordering genes. Differentially expressed genes (DEGs) between microglial subclusters were determined using the differentialGeneTest function. Genes with a *q*-value < 0.01 were considered significant and were subsequently selected and ranked by *q*-values using the setOrderingFilter function to guide the trajectory inference.

Dimensionality reduction was performed with the DDRTree algorithm using default settings *via* the reduceDimension function. Cells were then projected onto the trajectory manifold using the orderCells function, which also identified branching points. The trajectory root was assigned to the state predominantly composed of Sham-group microglia, represent the homeostatic baseline. Dynamic gene expression patterns along pseudotime and across branches were visualized using plot_cell_trajectory, plot_genes_in_pseudotime, plot_pseudotime_heatmap, and plot_genes_branched_heatmap functions.

### Cell-cell communication analysis

To investigate intercellular signaling networks, CellChat (v1.6.1) was applied to the normalized expression data from all clusters. The approach enabled the inference of infer ligand-receptor interactions and the calculation of communication probabilities based on a curated database of known interactions. Signaling pathways were ranked according to their overall communication probability, and key ligand-receptor pairs were visualized using network and circular plots.

### Statistical analysis and visualization

All statistical analyses and data visualizations were performed in R (v4.1.0). Visual representations—including UMAP embedding's, violin plots, heatmaps, and trajectory diagrams—were generated using ggplot2 (v3.4.4), ComplexHeatmap (v2.15.1), and plotting functions from the Monocle2 package. Unless otherwise specified, statistical significance was defined as *p* < 0.05.

### Cell culture

BV2 microglial cells (Servicebio, STCC20009P) were cultured in DMEM supplemented with 10% FBS and 1% penicillin-streptomycin. Cells were placed in a humidified incubator at 37°C with 5% CO_2_. When cultures reached 70%−80% confluence, cells were switched to low serum (1% FBS) for 24 h; followed by stimulation with 1, 5, and 10 ug/ml of LPS (Servicebio, GC205009) and 25, 50, and 100 ng/ml of OSM recombinant protein (MedChemExpress, HY-P7274) for 24 h, prior to downstream experiments.

### CCK8

Cell viability was assessed using the Cell Counting Kit-8 (G4103, Servicebio) following the manufacturer's protocol. Cells were seeded into 96-well plates and subjected to indicated treatments. At the specified time points, 10 μL CCK-8 solution was added to each well and incubated for 1–2 h at 37 C. Absorbance was measured at 450 nm using a microplate reader, and cell viability was calculated relative to control groups. All experiments were performed in triplicate.

### Western blot

Protein were extract and quantified from cultured cells. Equal amount of total proteins were separated by SDS-PAGE and transfered onto PVDF membranes. Membrane were blocked at room temperature with 1% fetal bovine serum (BSA) for 2 h. Membranes were then incubated overnight in a refrigerator at 4 C, with the following primary antibodies: rabbit anti-IL-6 antibody (Servicebio, GB11117), rabbit anti-TNF-α antibody (Servicebio, GB12188), rabbit anti-β-actin antibody (Servicebio, GB11001).

### Statistical analysis

All statistical analysis were performed using GraphPad Prism 9. Data are presented as mean ± standard error of the mean (SEM). Difference between groups were evaluated using one-way analysis of variance (ANOVA), followed by Tukey's *post hoc* test. A *p*-value < 0.05 was considered statistically significant.

## Results

### Identification of cortical cell types and inflammatory activation following TBI

**Figure 1:**
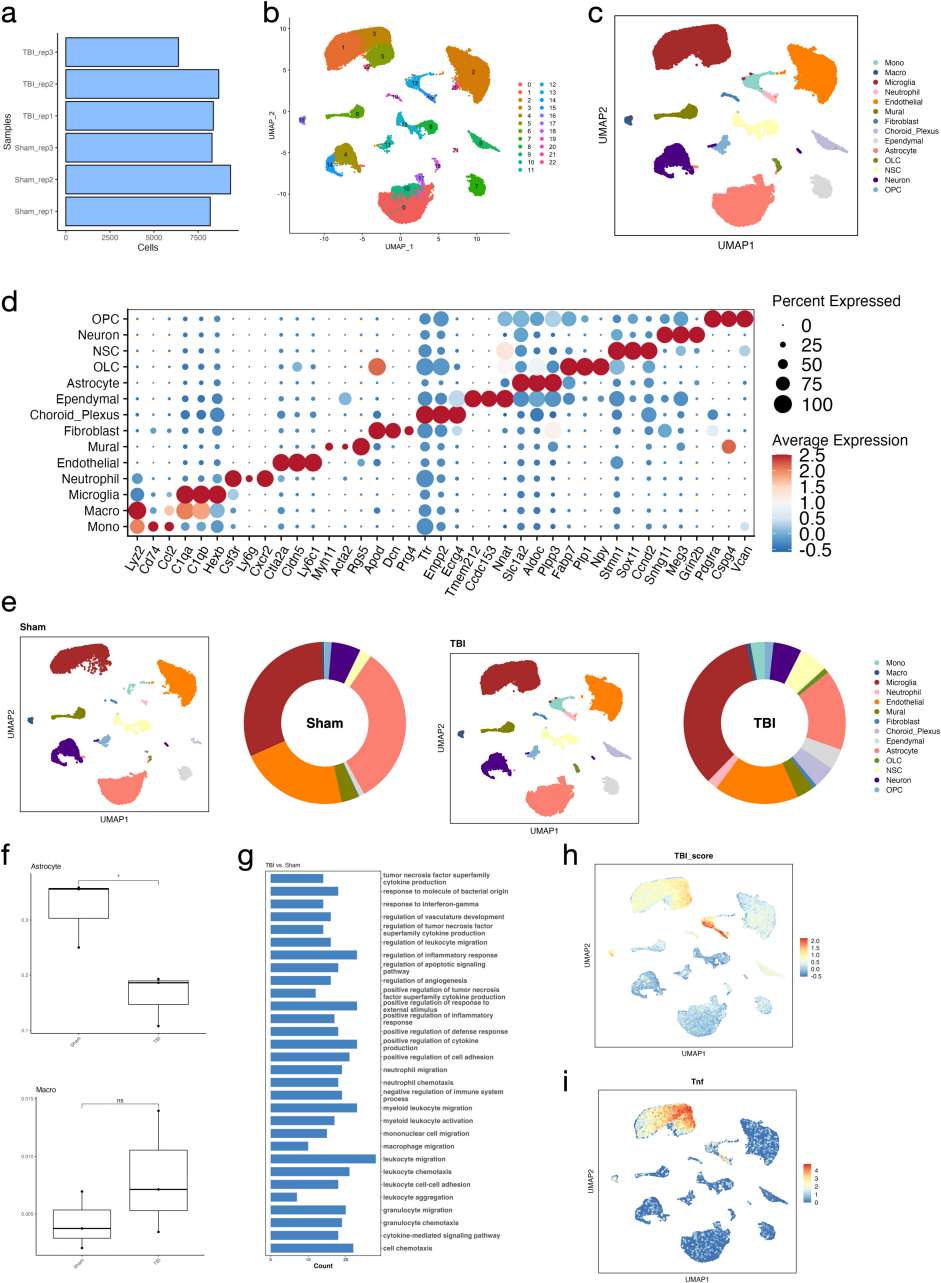
**(a)** cell number of each sample; **(b)** UMAP of each cluster; **(c)** UMAP of each cell type; **(d)** dot plot of each cell type markers; **(e)** UMAP of each condition and circle bar plot of each cell type; **(f)** box plot shows the percentage of astrocyte and macroglia; **(g)** bar plot of GO analysis results; **(h)** UMAP shows the level of TBI score; **(i)** UMAP shows the expression of *Tnf*.

To characterize the cellular landscape of the mouse cortex after traumatic brain injury (TBI), we reanalysis the single-cell RNA sequencing (scRNA-seq) data from three TBI samples and three sham samples obtained from GEO database (accession GSE290150). Low-quality cells were removed based on the following criteria: mitochondrial genes content >20%, fewer than 200 detected RNA features, and hemoglobin genes content >1%. Doublets were also identified and excluded. The six cortical samples were then integrated using Harmony and cell clusters were determined *via* a graph-based clustering method (see Materials and Methods). All the six samples were grouped into 22 clusters (Figures 1a–c).

These clusters were effectively annotated to major cortical cell types based on conventional marker genes expression (Figures 1c, d, Supplementary Figure S1a). The identical cell types included: Monocytes (Mono; *Lyz2, Cd74, and Ccl2*), Macrophages (Macro; *C1qa, and C1qb*), Microglia (Microglia; *C1qa, C1qb, and Hexb*), Neutrophils (Neutrophil; *Csf3r, Ly6g, and Cxcr2*), Endothelial cells (Endothelial; *Ctla2a, Cldn5, and Ly6c1*), Mural cells (Mural; *Myh11, Acta2, and Rgs5*), Fibroblasts (Fibroblast; *Apod, Dcn, and Prg4*), Choroid Plexus (Choroid Plexus; *Ttr, Enpp2, and Ecrg4)*, Ependymal cells (Ependymal; *Tmem212, Ccdc153, and Nnat*), Astrocytes (Astrocyte; *Slc1a2, Aldoc, and Plpp3*), Oligodendrocyte lineage cells (OLC; *Fabp7, Plp1, and Npy*), Neural stem cells (NSC; *Stmn1, Sox11, and Ccnd2*), Neurons (Neuron; *Snhg11, Meg3, and Grin2b*), and Oligodendrocyte precursor cells (OPC; *Pdgfra, Cspg4, and Vcan*) (29, 32) (Supplementary Figure S1A). Importantly, markers such as *Lyz2, Cd74, C1qa, C1qb, C1qb, Ccl2*, and *Hexb*, were used to differentiate between the myeloid-derived monocytes, macrophages, and the brain-resident microglia. Clusters corresponding to the same cell type were merged, resulting in a final set of 14 different cell populations in the mouse cortex (Figures 1c, d).

At this point, cells were separated into the sham and TBI groups according to their sample origin and the relative proportion of each cell type was compared between the two groups (Figures 1e, f). In the sham group, astrocytes (32.34%) and microglia (31.15%) represented the dominant cell populations. Following TBI, the proportion of microglia showed no significant changes (34.05%, *p* > 0.05), whereas the proportion of astrocytes was significantly reduced to (16.10%, *p* < 0.05; Figures 1 e–h).

Differentially gene expression analysis between TBI and Sham groups was performed followed by gene set enrichment analysis (Figure 1g). This analysis revealed significant enrichment of genes associated with immune and inflammatory response. Enriched biological process included response to interferon-gamma, tumor necrosis factor superfamily cytokine production, inflammatory response regulation, leukocyte migration and chemotaxis, positive regulation of cell adhesion, and leukocyte activation. These findings indicate the presence of a coordinated and robust immune response within the cerebral cortex according to moderate TBI. Simultaneously, the enrichment of pathways related to leukocyte migration, leukocyte chemotaxis, and cell adhesion suggest effective attraction and infiltration of immune cells into the injured part of the cortex, involving not only resident microglia but also potentially peripheral immune cell populations that contribute to the inflammatory microenvironment after TBI.

We next calculated a TBI-associated gene signature score (TBI score) based on the differing expressed genes identified in the TBI group (Figure 1h). This particular analysis indicated that the microglia, neutrophils, monocytes, and macrophages exhibited relatively high TBI score among all other cell types. Moreover, elevated expression of Tnf was observed in microglia (Figure 1i). These results suggest that although their overall percentage of microglia did not change substantial, microglia remain highly active and involved in the inflammatory response at the cortex following TBI.

### Microglial subclustering reveals a shift from homeostatic to disease-associated states following TBI

**Figure 2:**
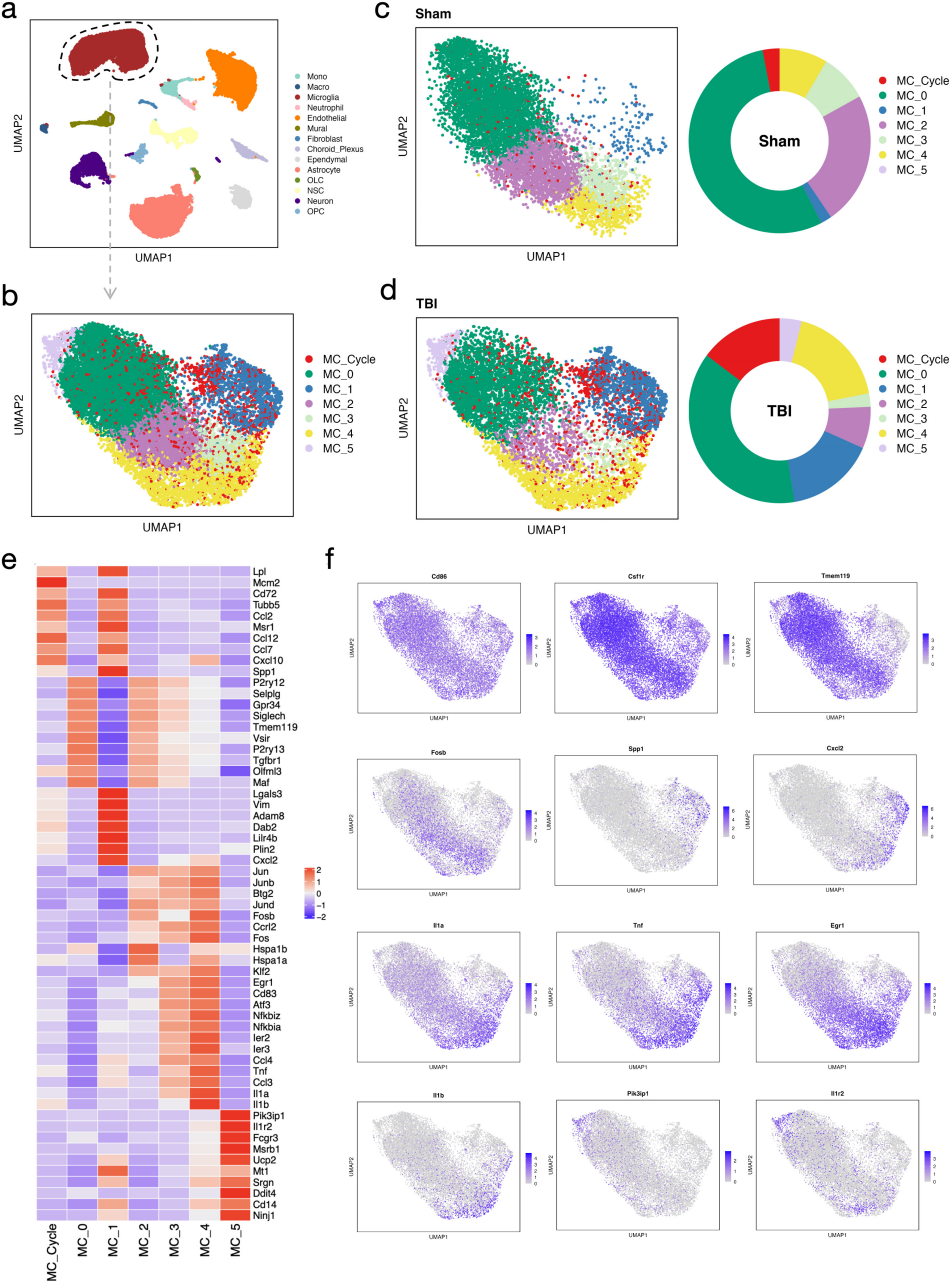
**(a)** UMAP shows the macroglia cells of all samples; **(b)** UMAP shows the macroglia subclusters of all samples; **(c)** UMAP and circle bar plot shows the macroglia subclusters in sham condition; **(d)** UMAP and circle bar plot shows the macroglia subclusters in TBI condition; **(e)** heat map shows the makers of each macroglia subcluster; **(f)** UMAP shows the makers of each macroglia subcluster.

Following isolation of the microglia, subclustering analysis identify seven distinct subpopulations of transcriptomically different cells (Figures 2a, b). Among these, cluster C2 and C0 were the most common microglia in the sham group. After TBI, the proportions of the homeostatic subpopulations C0 and C2 were markedly reduced, accompanied by an increase in several activated subpopulations, including the proliferative population (MC Cycle) and C1, C4, and C5 clusters (Figures 2c, d). Differential gene expression analysis further confirmed that these subpopulations exhibited distinct transcriptional signatures (Figures 2e, f). Notably, The homeostatic microglial marker *Tmem119* was highly expressed in clusters C0, C2, and C3, whereas its expression was markedly reduced in the MC Cycle, C1, C4, and C5 clusters. This observation is essential, because Transmembrane protein 119 (TMEM119) is widely recognized as a marker of homeostatic microglia. Previous studies have demonstrated that the overexpression of *TMEM119* enhances phagocytic capacity of microglia, whereas degradation of *TMEM119* disrupts microglia homeostasis and promotes the transition toward disease-associated states (33).

Further examination of the clusters revealed that C1 and C4 displayed features consistent with disease-associated microglia (DAM) including elevated expression of *Spp1* and *Tnf* . Osteopontin (SPP1) has previously been associated with enhanced phagocytic activity in mouse models of the Alzheimer's disease and its expression has been strongly correlated with synaptic loss. Moreover, cluster C1 showed high expression of *Cxcl2*, a secreted immunoregulatory factor that involved in inflammatory reponses (34). Cluster C4 was characterized by strongly expression of the pro-inflammatory cytokine Il1a and Il1b, whereas cluster C5 exhibited elevated expression of *Il1r2*, which encodes the decoy receptor Il-1R2 (CD121b) together with Pik3ip1, an inhibitor of phosphatidylinositol 3-kinase signaling.

**Figure 3:**
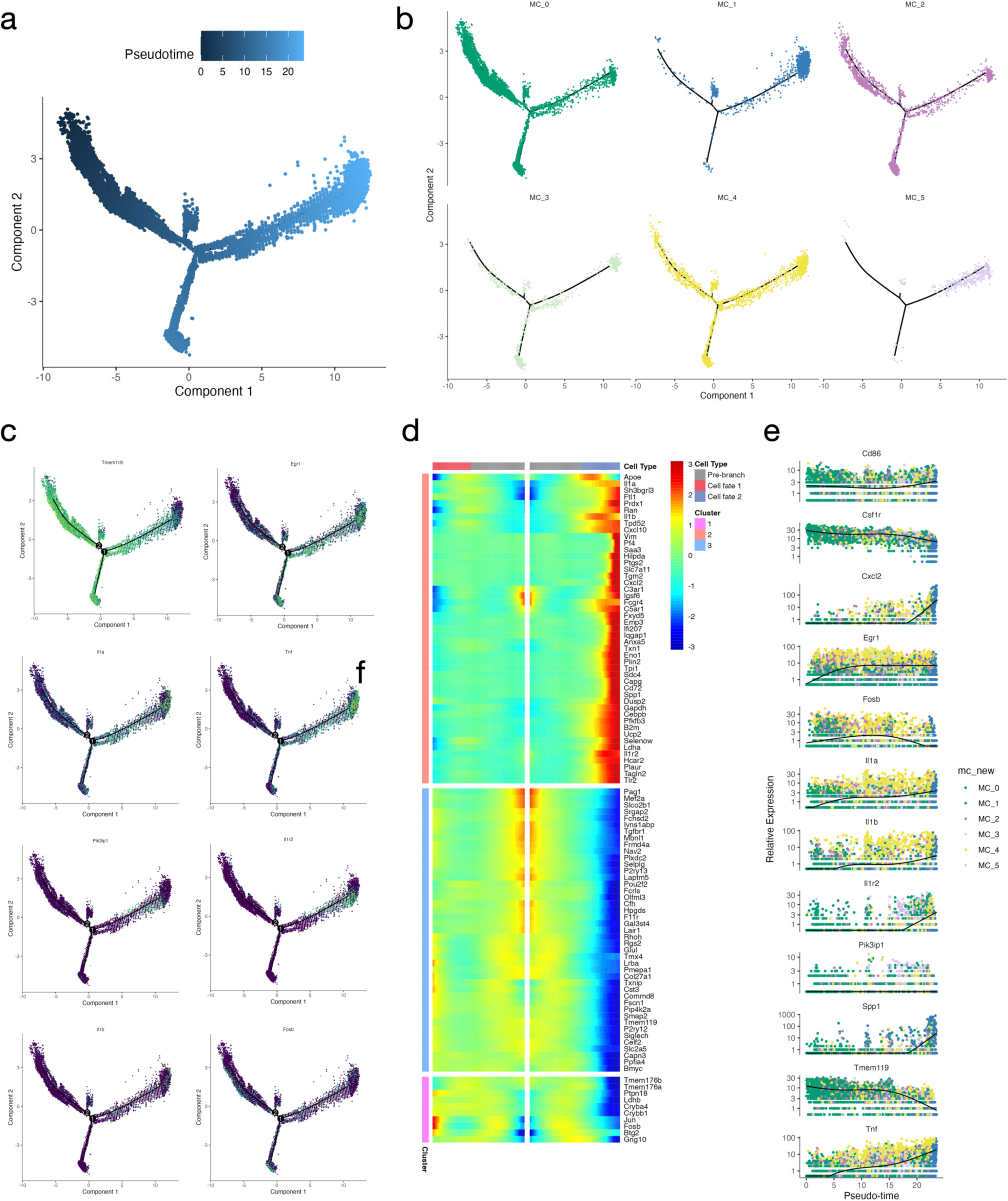
**(a)** pseudotime trajectory show the levels of pseudotime score; **(b)** pseudotime trajectory shows the levels of pseudotime score in each macroglia subcluster; **(c–e)** pseudotime trajectory shows the expression of selected genes.

The observed alteration in microglial subpopulation composition indicate a clear transition from homeostatic to disease-associated states in the cerebral cortex following TBI. This dynamic phenomenon was further supported by trajectory inferences analysis, which reconstructed the state transitions of microglia after brain injury (Figure 3a). Homeostatic microglia populations (C0, C2, and C3) were primarily distributed along the left and lower regions of the trajectory, whereas disease-associated microglia popuations (C1, C4, and C5) were predominantly localized within the central and right regions of the trajectory (Figure 3b).

Mapping the marker genes of each subpopulation onto the pseudotime trajectory revealed a high concordance between gene expression patterns and cellular state distribution (Figure 3c). The trajectory primarily originated from cluster C0, and with progression along pseudotime, microglia gradually transitioned toward inflammatory phenotypes, represented by clusters C1, C4, and C5. Temporal gene expression analysis demonstrated that Tmem119 expression was highest at the early stages of the trajectory and gradually declined over pseudotime. On the other hand, the expression levels of inflammatory-associated genes, including*Tnf, Spp1, Il1a, Il1b*, and *Cxcl2*, gradually increased along the trajectory direction (Figures 3d, e). Notably, the canonical microglial markers *Cd86* and *Csf1r* maintained relatively stable throughout the transitionprocess, confirming that microglia preserved their lineage identity during despite undergoing substantial phenotypic changes.

### Proliferating microglia shift towards pro-inflammatory phenotypes after TBI

**Figure 4:**
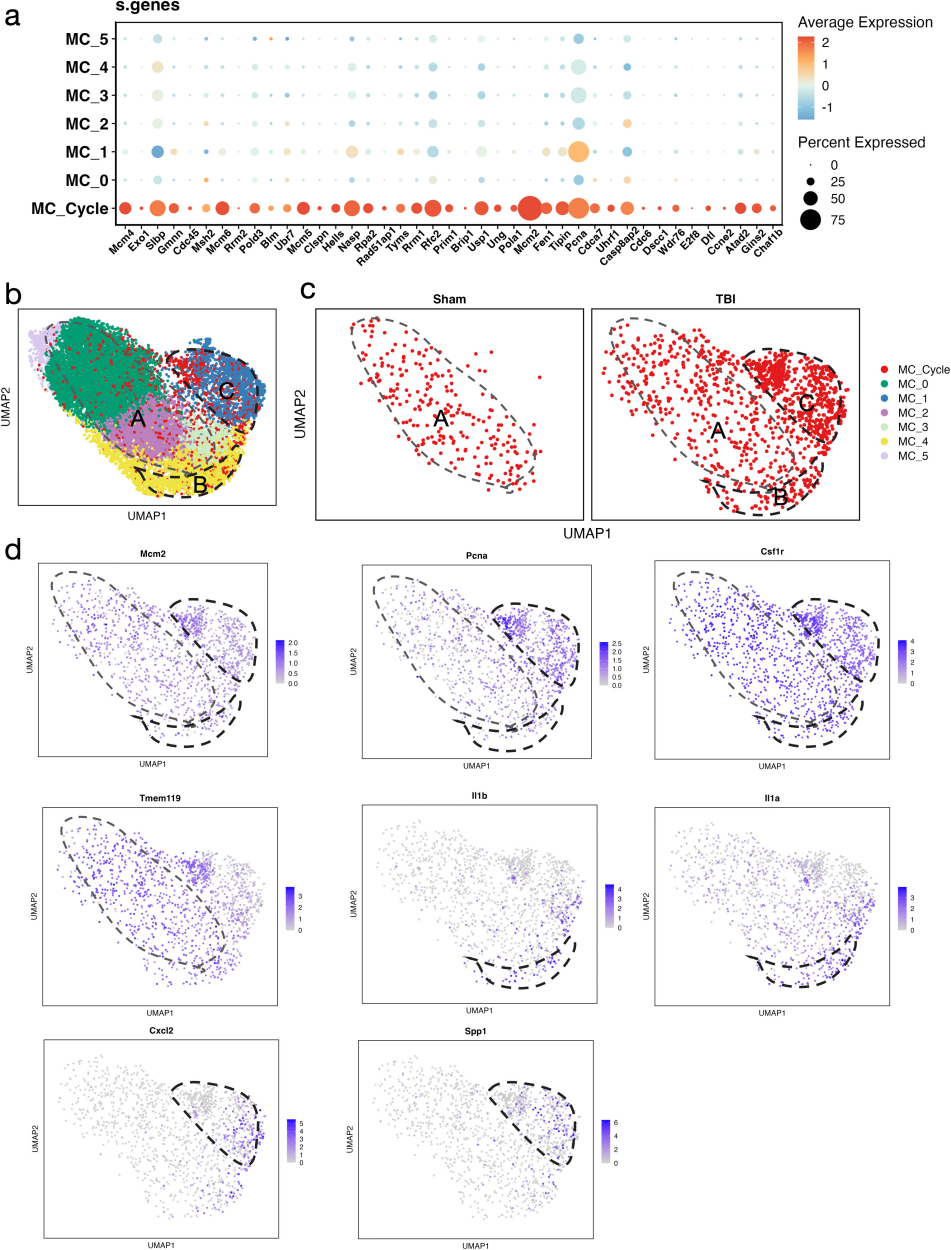
**(a)** dot plot shows the expression of S-phase genes in each macroglia subcluster; **(b, c)** UMAP shows A-C region of proliferating macroglia cells; **(d)** UMAP shows the expression of selected genes in proliferating macroglia cells.

The increased proportion of the MC Cycle subpopulation following TBI prompted further investigation into microglial proliferation. In these cells, the genes linked with the mitotic S-phase, e.g., *Mcm4, Mcm2, Ubr7*, and *Pcna*, exhibit high expression (Figure 4a). Previous studies have demonstrated the remarkable regenerative ability of microglia and have suggested that proliferating microglia are primarily derived from surviving microglia. This implies that the M1-polarized microglia proliferation in the mouse cerebral cortex may result not only from phenotypic conversion of the M2-polarized microglia but also from active proliferation of resident microglial populations (35).

To further characterize proliferating microglia, the MC Cycle subpopulation was isolated and subjected to re-dimensionality reduction and clustering analysis followed by visualization using UMAP plot. The UMAP projection highlighted the transcription relationships and the heterogeneity of the proliferating microglia. The two-dimensional representation of MC Cycle cell demonstrated a significant overlap with the other microglial subpopulations, which suggests that proliferating cells are transcriptionally associated with specific microglial states rather than forming a distinct separate group. Distinct gene expression patterns enabled further classification of proliferating MC Cycle cells and allowed comparison of their distribution between Sham and TBI conditions (Figures 4b, c). Based on this analysis, MC Cycle cells were divided into three major regions. Region A primarily consisted of proliferating homeostatic microglia, characterized by high *TMEM119* expression; region B was mainly composed of proliferating C4-like microglia, with elevatedexpression of pro-inflammatory cytokines *Il1a* and *Il1b*. Region C predominantly contained proliferating C1-like microglia characterized by high levels of *Spp1* and *Tnf* . The microglial marker *Csf1r*, together with proliferation-associated genes *Mcm2* and *Pcna*, was consistently expressed across all regions, confirming their identity as proliferating microglia. Comparison of the MC Cycle between Sham and TBI groups revealed a significant shift in the distribution of proliferating microglia phenotype (Figure 4d). In the Sham group, proliferating microglia were predominantly associated with homeostatic states. By contrast, TBI group demonstrated a significant proliferation of the M1-like microglia, with the C1-like subpopulation representing the dominant proliferative phenotype. These findings suggest that the post-TBI inflammatory milieu not only stimulates microglial proliferation but also skews it toward a pro-inflammatory phenotype, thereby potentially exacerbating neuroinflammation and contributing to secondary injury progression.

### TBI rewires microglial communication networks and enhances pro-inflammatory signaling

**Figure 5:**
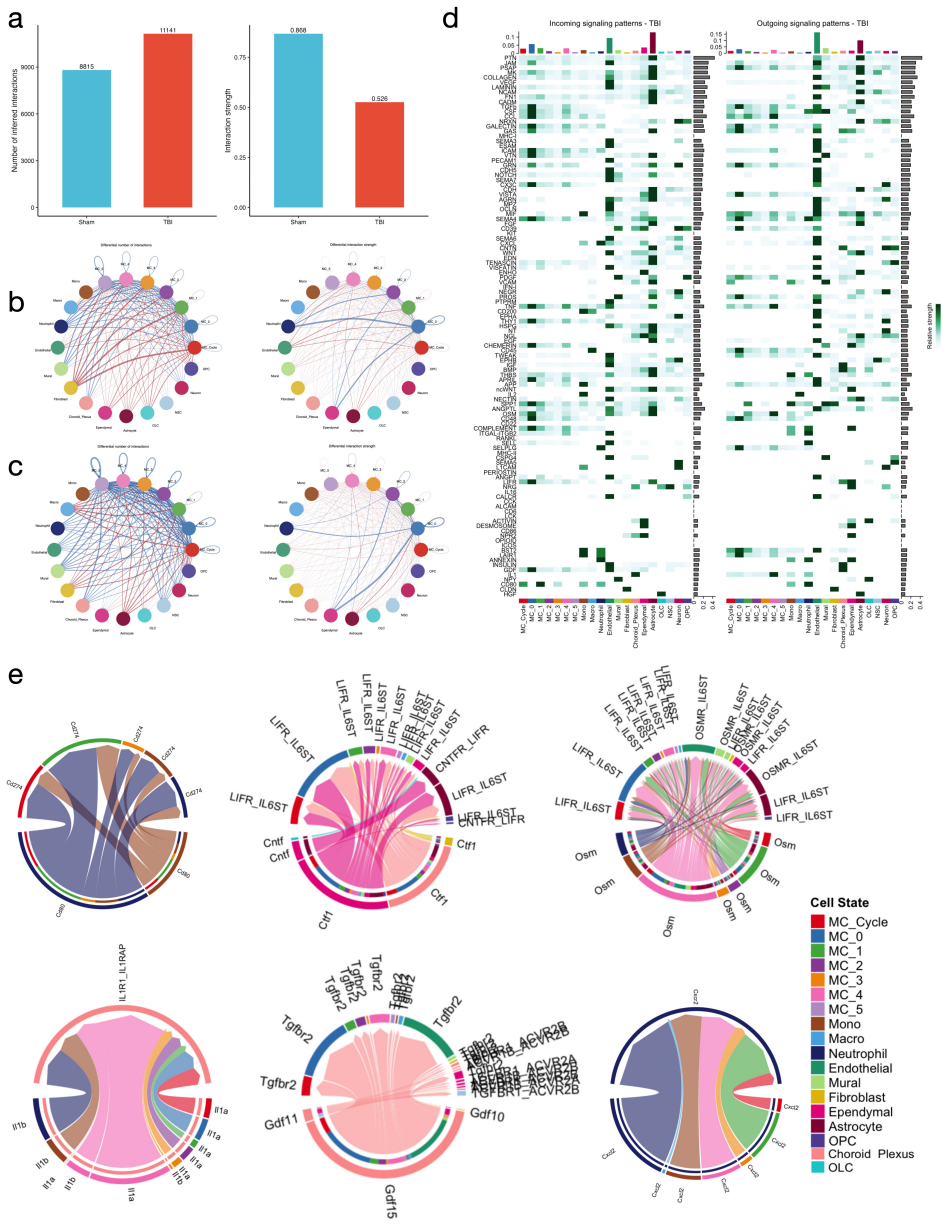
**(a)** bar plots show the number and strength of interred interactions; **(b)** circle plot shows the number and strength of interred interactions to macroglia subclusters; **(c)** circle plot shows the number and strength of interred interactions from macroglia subclusters; **(d)** heatmap shows the incoming and outgoing signaling pattern in TBI; **(e)** chord plot shows the interred interactions of selected pathway.

To investigate alterations in intercellular communication following traumatic brain injury, we performed a systematic comparative cellchat analysis between the sham and TBI groups. Although a greater number of interaction pathways was detected after TBI, the overall communication strength was markedly reduced, suggesting that increased cellular heterogeneity may compromisecommunication efficiency within the injured cortex (Figure 5a). Interestingly, distinct microglial groups exhibited different communication patterns.M2-like microglia (C0, C2) displayed less signaling strength, whereas M1-like groups (C1, C4) showed enhanced outgoing communication, indicating a shift in inflammatory network control to pro-inflammatory phenotypes (Figures 5b, c). Mechanistically, several signaling pathways were significantly activated after TBI. Pathways involving CD80, LIFR, OSM, IL-1, GDF, and CXCL, signaling were particularly enriched in M1-like microglia (Figures 5d, e). In particular, C1 microglia interacted with neutrophils through the CD80–CD274 immune checkpoint pathways, while both classes of microglia recruited neutrophils using the Cxcl2–Cxcr2 chemotactic axis. At the same time, these cells were subjected to positive regulators through the LIFR receptor mediated by factors such as Osm, Ctf1, and Cntf. In addition, Tgfbr2-expressing C1 and C4 microglia were responsive to choroid plexus cells *via* secretion of GDF15, creating a pathway of microglial and transdifferentiated cells. Collectively, these findings demonstrate extensive remodeling of cellular communication networks within the post-TBI neuroinflammatory microenvironment. In particular, microglia-neutrophil interaction and bidirectional communication between microglia and choroid plexus cells merged as signaling modules that may contributing to the maintenance of the inflammatory status after TBI. These observations provide additional insight into the mechanisms underlying secondary injury progression following traumatic brain injury.

### OSM induces inflammatory activation and phenotypic remodeling in microglia

**Figure 6:**
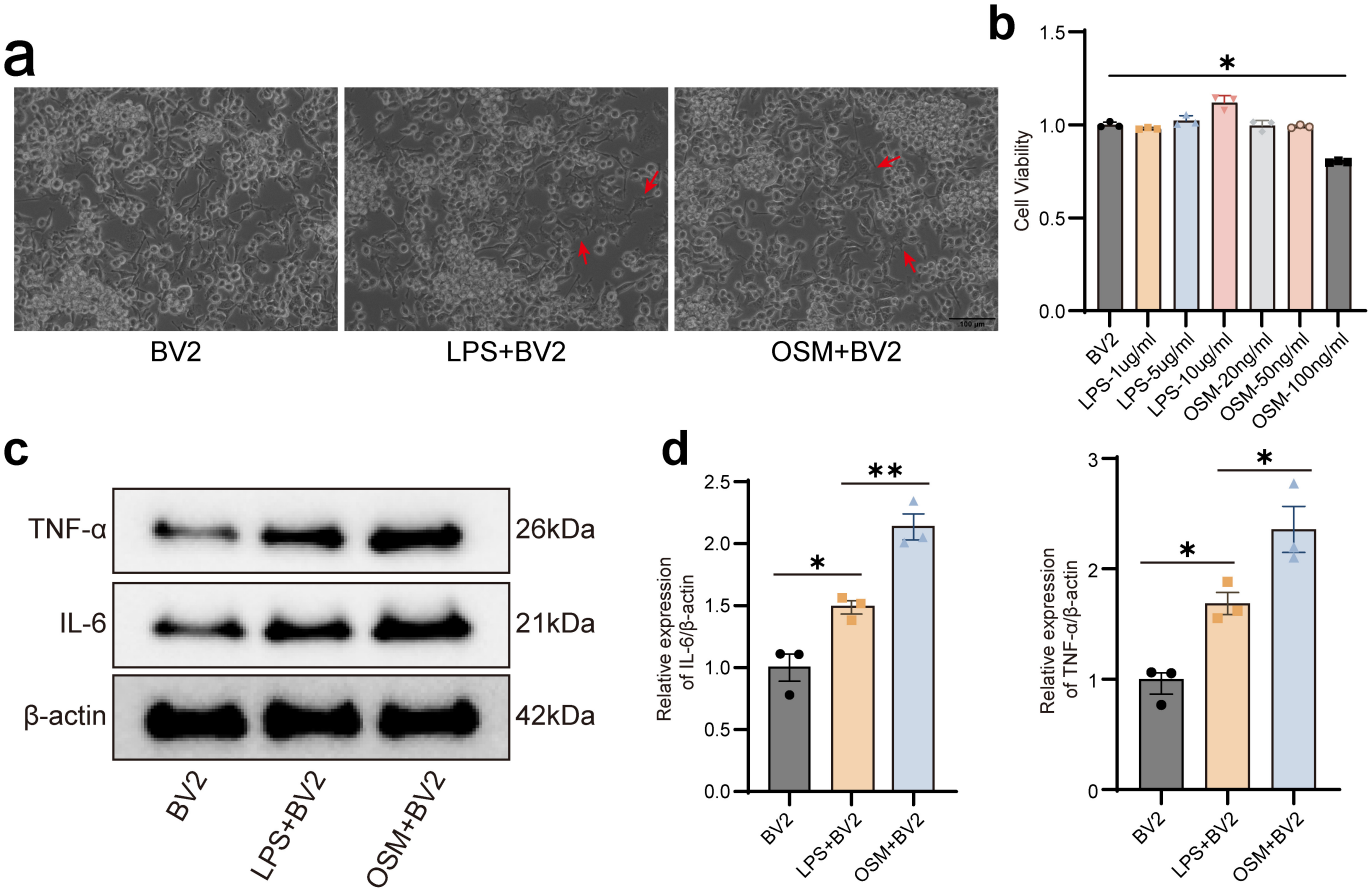
**(a)** Representative bright-field images showing morphological changes in BV2 microglia following recombinant OSM stimulation. Compared with untreated cells, OSM-treated BV2 cells exhibited enlarged soma, shortened processes, and a more amoeboid-like morphology, consistent with microglial activation. **(b)** CCK8 assay detects the cell viability of each group; **(c, d)** The representative images and quantitative analysis of protein expression of IL-6, TNF-α, and β-actin detected by Western blot; Data were expressed as the mean±SEM, *P < 0.05, **P < 0.01, vs. indicated groups *n* = 3 for each group.

To provide preliminary experimental validation for the computationally predicted signaling network, OSM was selected for follow-up analysis because CellChat analysis identified the OSM–LIFR axis as one of the prominently enriched signaling pathways associated with inflammatory microglial subsets after TBI. Furthermore, as a soluble cytokine with established immunomodulatory role in glial biology, OSM represents a suitable candidate for reductionist *in vitro* validation. Morphologically, resting BV2 cells exhibited a ramified or spindle-like appearance with elongated cellular processes. Following, OSM stimulation induced marked morphological changes characterized by enlarged soma, shortened processes, and a more amoeboid-like phenotype, features consistent with activated microglial (Figure 6a). To determine an appropriate non-cytotoxic concentration for subsequent experiments, BV2 viability was evaluated after treatment with increasing concentrations of recombinant OSM. CCK-8 analysis demonstrated that OSM concentration of 25–50 ng/mL produced minimal effects on cell viability, whereas treatment with 100 ng/mL resulted in a modest reduction in viability, indicating concentration-dependent cellular stress at higher doses (Figure 6b). Based on these results, 50 ng/mL was selected as the working concentration for downstream assays. At this concentration, OSM markedly increased the expression of pro-inflammatory mediators, including TNF-α and IL-6, compared with untreated controls (Figures 6c, d). Together, these findings demonstrate that OSM is sufficient to induce inflammatory activation in microglia, supporting the biological relevance of the OSM-associated signaling pathways predicted by single-cell communication analysis.

## Discussion

Compared with the prior study, our scRNA-seq analysis enabled a more refined characterization of microglial states and revealed a pronounced transition toward disease-associated and relevant phenotypes after TBI. Moreover, our cell-cell communication analysis identified severalsignaling pathways that may coordinate neuroinflammation reponses and contribute to the development of secondary injuries.

We observed substantial redistribution of microglial subpopulations after traumatic brain injury (TBI). Seven distinct microglial clusters were identified. While the homeostatic subsets (C0 and C2) were predominant under sham conditions, they were markedly reduced post-injury. In contrast, several activated subpopulations expanded significantly, including the proliferating microglia (MC Cycle) and the pro-inflammatory or disease-associated clusters (C1, C4, and C5). To further characterize these phenotypic transitions, pseudotemporal trajectory analysis was performed to reconstruct the dynamic progression of microglial states. This analysis revealed a gradual transcriptomic transition from homeostatic microglia toward disease-associated phenotypes. This transition was characterized by the progressive downregulation of the homeostatic marker Tmem119 together with increase expression of inflammatory cytokines and markers, including *Tnf, Spp1, Il1a, Il1b*, and *Cxcl2*.

Previous histological studies have reported a nearly 20-fold increase in microglial proliferation during the acute phase of CCI-induced TBI (36). Consistent with these observations, our scRNA-seq analysis identified a distinct proliferative population, reflecting robust microgliosis after injury. Importantly, trajectory analysis further reveals the underlying cellular kinetics: this post-TBI microgliosis does not merely expand the size of the microglial pool but actively promotes its composition toward pro-inflammatory and neurotoxic trajectories, potentially sustaining the secondary injury environment.

The expansion of MC Cycle subpopulation prompted further investigation into microglia. The upregulation of S-phase genes (*Mcm4, Mcm2, Ubr7, and Pcna*) confirmed the actively proliferative state of these cells. Notably, our subset analysis demonstrated that proliferating microglia under TBI conditions were biased mainly toward M1-like phenotypes (especially C1-like), whereas homeostatic proliferating microglia dominated in sham controls. These findings indicate that the post-TBI inflammatory milieu not only stimulates microglial proliferation but also directs it toward pro-inflammatory trajectories, potentially amplifying neuroinflammation through continuous replenishment of activated microglia.

Our CellChat analysis further revealed a extensive remodeling of intercellular communication networks after TBI. Although signaling diversity increased, the overall communication strength reduced, possibly reflecting disruption of coordinated signaling caused by increased cellular heterogeneity. Importantly, signaling dominance shifted from M2-like microglia (C0 and C2) toward M1-like subsets (C1 and C4), highlighting their enhanced influence within the inflammatory network. Several key pathways were specifically upregulated in M1-like microglia, including LIFR, IL1, CXCL, and CD80 signaling axes.

In particularly, C1 and C4 microglia received regulatory signals through the LIFR receptor activated by ligands such as *Osm, Ctf1*, and *Cntf*. The LIF/LIFR signaling axis is known to activate oncogenic signaling pathways including JAK/STAT3, MAPK, and AKT/mTOR in cancer contexts (37). Within the TBI milieu, our findings suggest that M1 microglia may potentially mediate astrocyte dysfunction by Osm-Lifr signaling, possibly contribute to inflammatory amplification. At the same time, microglia signaled to the choroid plexus through IL1-related pathways, whereas choroid plexus cells secreted *GDF15* that targeted *Tgfbr2*-expressing C1 and C4 microglia. This bidirectional microglia-choroid plexus circuit, particularly involving IL1 signaling, suggests a novel mechanism for sustaining neuroinflammation; specifically, choroid plexus inflammation can disrupt barrier function and promote leukocyte infiltration (38). In addition, both C1 and C4 microglia recruited neutrophils through the Cxcl2–Cxcr2 chemotactic signaling axis. *Cxcl2* is a chemokine closely associated with immunoregulation and inflammatory regulation (34), and neutrophil infiltration may further exacerbate parenchymal inflammation. Interestingly, neutrophils expressed *Cd80*, which interacted with *Cd274* on microglia. The CD80–CD274 (PD-L1) immune checkpoint pathways has previously been implicated in the regulation of neuroinflammation, and PD-1 signaling has been shown to limit CNS inflammation in experimental stroke (39). In the present TBI model, however, the interaction between neutrophil and microglia through the Cd80–Cd274 may instead contribute to sustained M1 polarization by overriding inhibitory immune checkpoint signaling, thereby reinforcing pro-inflammatory microglial activation.

Collectively, these findings highlight a complex cellular network in which M1-like microglia occupy a central regulatory role in post-TBI neuroinflammation. These cells participate in multidirectional signaling interactions by receiving and providing activation signals through autocrine and paracrine pathways; recruiting peripheral immune cells *via* chemokine secretion; influencing barrier function at the choroid plexus; and interacting with neutrophils through the Cd80–Cd274 axis that may reinforce their pro-inflammatory phenotype.

Our study has several limitations that should be acknowledged. First, the present analyzed was based on a publicly available scRNA-seq dataset. Validation using independent cohorts and complementary approaches such as spatial transcriptomics would further strengthen the reliability of our findings. In addition, the mechanistic roles of the identified signaling pathways, particularly the functional effects of Osm-Lifr signaling in astrocytes and Cd80–Cd274 interaction between neutrophils and microglia, require further the experimental investigation using genetic and pharmacological methods in TBI models.

We also acknowledge the limitations associated with interpreting microglial activation using the traditional M1/M2 polarization framework. Recent single-cell studies suggest that microglia do not exist in rigid defined binary states but instead exhibit a continuous activation spectrum with significant functional heterogeneity. Although M1/M2-related markers were used in the present study to facilitate the initial cluster characterization, the observed phenotypes in our TBI model likely represent specific states within a broader disease-associated microglia (DAM) or injury-associated activation landscape. Future studies intergrading high-dimensional spatial proteomics and functional assays will be essential for further refining these transitional states and elucidating the full complexity of microglial plasticity during neurodegeneration.

Despite these limitations, our study provide a fruitful source of information on cortical cellular responses to TBI at the single-cell level. We defined the dynamically spectrum of microglial states, identified their proliferative dynamics, and uncovered an intercellular communication network associated with neuroinflammation. The signaling pathways identified in this study, particularly the LIFR, IL1, CXCL, and CD80 signaling pathways, may represent promising targets for further immunomodulatory strategies aimed at reducing secondary injury following traumatic brain injury.

## Data Availability

The original contributions presented in the study are included in the article/Supplementary material, further inquiries can be directed to the corresponding author.

## Supplementary material

Supplementary Figure 1 Dot shows the expression of cell type markers in each clusters.

Supplementary Figure 2 Heatmap shows the incoming and outgoing signaling pattern in Sham group.

## References

[1] Andelic N. The epidemiology of traumatic brain injury. Lancet Neurol. (2013) 12:28–9. doi: 10.1016/S1474-4422(12)70294-6 23177533

[2] Siebold L Obenaus A Goyal R. Criteria to define mild, moderate, and severe traumatic brain injury in the mouse controlled cortical impact model. Exp Neurol. (2018) 310:48–57. doi: 10.1016/j.expneurol.2018.07.004 30017882

[3] Dewan MC Rattani A Gupta S Baticulon RE Hung Y Punchak M . Estimating the global incidence of traumatic brain injury. J Neurosurg. (2019) 130:1080–97. doi: 10.3171/2017.10.JNS17352 29701556

[4] Taylor CA Bell JM Breiding MJ Xu L. Traumatic brain injury-related emergency department visits, hospitalizations, and deaths - United States, 2007 and 2013. MMWR Surveill Summ. (2017) 66:1–16. doi: 10.15585/mmwr.ss6609a1 28301451 PMC5829835

[5] Khellaf A Khan DZ Helmy A. Recent advances in traumatic brain injury. J Neurol. (2019) 266:2878–89. doi: 10.1007/s00415-019-09541-4 31563989 PMC6803592

[6] Girgis F Pace J Sweet J Miller JP. Hippocampal neurophysiologic changes after mild traumatic brain injury and potential neuromodulation treatment approaches. Front Syst Neurosci. (2016) 10:8. doi: 10.3389/fnsys.2016.00008 26903824 PMC4746250

[7] Bielanin JP Metwally SAH Paruchuri SS Sun D. An overview of mild traumatic brain injuries and emerging therapeutic targets. Neurochem Int. (2024) 172:105655. doi: 10.1016/j.neuint.2023.105655 38072207

[8] Janowitz T Menon DK. Exploring new routes for neuroprotective drug development in traumatic brain injury. Sci Transl Med. (2010) 2:27rv21. doi: 10.1126/scitranslmed.3000330 20393189

[9] Gruenbaum SE Zlotnik A Gruenbaum BF Hersey D Bilotta F. Pharmacologic neuroprotection for functional outcomes after traumatic brain injury: a systematic review of the clinical literature. CNS Drugs (2016) 30:791–806. doi: 10.1007/s40263-016-0355-2 27339615 PMC5116376

[10] Barker S Paul BD Pieper AA. Increased risk of aging-related neurodegenerative disease after traumatic brain injury. Biomedicines. (2023) 11:1154. doi: 10.3390/biomedicines11041154 37189772 PMC10135798

[11] Gaetz M. The neurophysiology of brain injury. Clin Neurophysiol. (2004) 115:4–18. doi: 10.1016/S1388-2457(03)00258-X 14706464

[12] Simon DW McGeachy MJ Bayir H Clark RS Loane DJ Kochanek PM. The far-reaching scope of neuroinflammation after traumatic brain injury. Nat Rev Neurol. (2017) 13:171–91. doi: 10.1038/nrneurol.2017.13 28186177 PMC5675525

[13] Anthonymuthu TS Kenny EM Bayir H. Therapies targeting lipid peroxidation in traumatic brain injury. Brain Res. (2016) 1640:57–76. doi: 10.1016/j.brainres.2016.02.006 26872597 PMC4870119

[14] Dorsett CR McGuire JL DePasquale EAK Gardner AE Floyd CL McCullumsmith RE. Glutamate neurotransmission in rodent models of traumatic brain injury. J Neurotrauma. (2017) 34:263–72. doi: 10.1089/neu.2015.4373 27256113 PMC5220558

[15] Bruns J Hauser WA. The epidemiology of traumatic brain injury: a review. Epilepsia (2003) 44:2–10. doi: 10.1046/j.1528-1157.44.s10.3.x 14511388

[16] Armstrong RC Mierzwa AJ Marion CM Sullivan G. M White matter involvement after TBI: Clues to axon and myelin repair capacity. Exp Neurol. (2016) 275(Pt 3):328–33. doi: 10.1016/j.expneurol.2015.02.011 25697845

[17] Corps KN Roth TL McGavern DB. Inflammation and neuroprotection in traumatic brain injury. JAMA Neurol. (2015) 72:355–62. doi: 10.1001/jamaneurol.2014.3558 25599342 PMC5001842

[18] Faden AI Wu J Stoica BA Loane DJ. Progressive inflammation-mediated neurodegeneration after traumatic brain or spinal cord injury. Br J Pharmacol. (2016) 173:681–91. doi: 10.1111/bph.13179 25939377 PMC4742301

[19] Davalos D Grutzendler J Yang G Kim JV Zuo Y Jung S . ATP mediates rapid microglial response to local brain injury in vivo. Nat Neurosci. (2005) 8:752–8. doi: 10.1038/nn1472 15895084

[20] Fourgeaud L Través PG Tufail Y Leal-Bailey H Lew ED Burrola PG . TAM receptors regulate multiple features of microglial physiology. Nature (2016) 532:240–4. doi: 10.1038/nature17630 27049947 PMC5358512

[21] Hanisch UK Kettenmann H. Microglia: active sensor and versatile effector cells in the normal and pathologic brain. Nat Neurosci. (2007) 10:1387–94. doi: 10.1038/nn1997 17965659

[22] Jassam YN Izzy S Whalen M McGavern DB El Khoury J. Neuroimmunology of traumatic brain injury: time for a paradigm shift. Neuron (2017) 95:1246–65. doi: 10.1016/j.neuron.2017.07.010 28910616 PMC5678753

[23] Loane DJ Kumar A. Microglia in the TBI brain: The good, the bad, and the dysregulated. Exp Neurol. (2016) 275(Pt 3): 316–27. doi: 10.1016/j.expneurol.2015.08.018 26342753 PMC4689601

[24] Witcher KG Bray CE Dziabis JE McKim DB Benner BN Rowe RK . Traumatic brain injury-induced neuronal damage in the somatosensory cortex causes formation of rod-shaped microglia that promote astrogliosis and persistent neuroinflammation. Glia (2018) 66:2719–36. doi: 10.1002/glia.23523 30378170 PMC7542609

[25] Myer DJ Gurkoff GG Lee SM Hovda DA Sofroniew MV. Essential protective roles of reactive astrocytes in traumatic brain injury. Brain (2006) 129:2761–72. doi: 10.1093/brain/awl165 16825202

[26] Heithoff BP George KK Phares AN Zuidhoek IA Munoz-Ballester C Robel S. Astrocytes are necessary for blood-brain barrier maintenance in the adult mouse brain. Glia (2021) 69:436–72. doi: 10.1002/glia.23908 32955153 PMC7736206

[27] Farina C Aloisi F Meinl E. Astrocytes are active players in cerebral innate immunity. Trends Immunol. (2007) 28:138–45. doi: 10.1016/j.it.2007.01.005 17276138

[28] Min KJ Yang MS Kim SU Jou I Joe EH. Astrocytes induce hemeoxygenase-1 expression in microglia: a feasible mechanism for preventing excessive brain inflammation. J Neurosci. (2006) 26:1880–7. doi: 10.1523/JNEUROSCI.3696-05.2006 16467537 PMC6793633

[29] Kerr NA Choi J Mohite SY Singh PK Bramlett HM Lee JK . Single cell RNA sequencing after moderate traumatic brain injury: effects of therapeutic hypothermia. J Neuroinflammation. (2025) 22:110. doi: 10.1186/s12974-025-03430-6 40251570 PMC12007139

[30] Xing J Ren L Xu H Zhao L Wang Z Hu G . Single-cell RNA sequencing reveals cellular and transcriptional changes associated with traumatic brain injury. Front Genet. (2022) 13:861428. doi: 10.3389/fgene.2022.861428 35846152 PMC9282873

[31] Arneson D Zhang G Ying Z Zhuang Y Byun HR Ahn IS . Single cell molecular alterations reveal target cells and pathways of concussive brain injury. Nat Commun. (2018) 9:3894. doi: 10.1038/s41467-018-06222-0 30254269 PMC6156584

[32] Marques S Zeisel A Codeluppi S van BD Mendanha FA Xiao L . Oligodendrocyte heterogeneity in the mouse juvenile and adult central nervous system. Science (2016) 352:1326–9. doi: 10.1126/science.aaf6463 27284195 PMC5221728

[33] Liu J Wang Z Liang W Zhang Z Deng Y Chen X . Microglial TMEM119 binds to amyloid-beta to promote its clearance in an Abeta-depositing mouse model of Alzheimer's disease. Immunity (2025) 58:1830–46 e1837. doi: 10.1016/j.immuni.2025.04.018 40373772

[34] Liu Y Xiao J Cai J Li R Sui X Zhang J . Single-cell immune profiling of mouse liver aging reveals Cxcl2+ macrophages recruit neutrophils to aggravate liver injury. Hepatology. (2024) 79:589–605. doi: 10.1097/HEP.0000000000000590 37695548 PMC10871588

[35] Huang Y Xu Z Xiong S Sun F Qin G Hu G . Repopulated microglia are solely derived from the proliferation of residual microglia after acute depletion. Nat Neurosci. (2018) 21:530–40. doi: 10.1038/s41593-018-0090-8 29472620

[36] Willis EF Kim SJ Chen W Nyuydzefe M MacDonald KPA Zanin-Zhorov A . ROCK2 regulates microglia proliferation and neuronal survival after traumatic brain injury. Brain Behav Immun. (2024) 117:181–94. doi: 10.1016/j.bbi.2024.01.004 38211634

[37] Viswanadhapalli S Dileep KV Zhang KYJ Nair HB Vadlamudi RK. Targeting LIF/LIFR signaling in cancer. Genes Dis. (2022) 9:973–80. doi: 10.1016/j.gendis.2021.04.003 35685476 PMC9170604

[38] Qin Q Fan L Zeng X Zheng D Wang H Li M . Mesenchymal stem cell-derived extracellular vesicles alleviate autism by regulating microglial glucose metabolism reprogramming and neuroinflammation through PD-1/PD-L1 interaction. J Nanobiotechnology. (2025) 23:201. doi: 10.1186/s12951-025-03250-z 40069859 PMC11895333

[39] Ren X Akiyoshi K Vandenbark AA Hurn PD Offner H. Programmed death-1 pathway limits central nervous system inflammation and neurologic deficits in murine experimental stroke. Stroke. (2011) 42:2578–83. doi: 10.1161/STROKEAHA.111.613182 21737801 PMC3164218

