# Supplementary material for: Single-cell RNA sequencing reveals microglial proliferative bias and neuroinflammatory communication reprogramming following traumatic brain injury
Source: Front Neurol. 2026 Jul 10;17:1789863. doi: 10.3389/fneur.2026.1789863 (PMC13404943; doi:10.3389/fneur.2026.1789863)
Supplement: Supplementary Figure 1 — Dot shows the expression of cell type markers in each clusters. [file Supplementary_file_1.pdf]

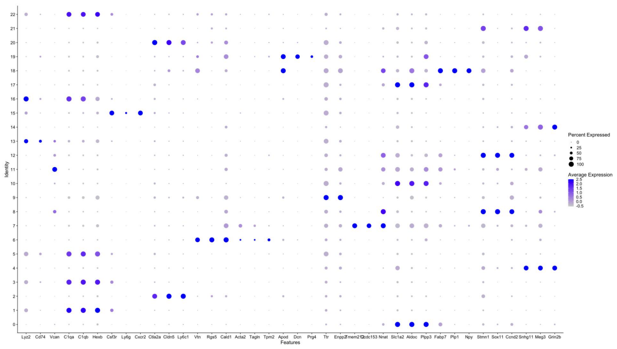

Figure S1 dot shows the expression of cell type markers in each clusters.

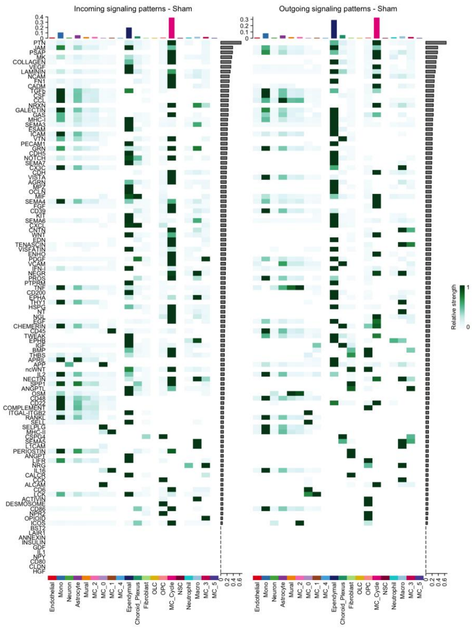

Figure S2 heatmap shows the incoming and outgoing signaling pattern in Sham group.
